# Decoding portal vein pulsatility: hemodynamic determinants in a post-hoc analysis of a prospective observational trial

**DOI:** 10.1186/s13613-025-01498-0

**Published:** 2025-06-14

**Authors:** Cosmin Balan, Bianca Morosanu, Antonia Fodoroiu, Vlad Dobre, Andrei Dumitrache, Robert Thomas Barbulescu, Liana Valeanu, Cornel Robu, Cristian Boros, Alexandru Nica, Adrian Wong, Francesco Corradi, Ioana Marina Grintescu, Serban-Ion Bubenek-Turconi

**Affiliations:** 1 1St Department of Cardiovascular Anesthesia and Intensive Care Medicine, Prof. Dr. C.C. Iliescu Institute for Emergency Cardiovascular Diseases, 022328 Bucharest, Romania; 2 Centre Hospitalier de Compiègne-Noyon, Noyon, France; 3 Department of Critical Care, King’s College Hospital, London, UK; 4 Department of Surgical, Medical, Molecular Pathology and Critical Care Medicine, University of Pisa, Pisa, Italy; 5 Department of Anaesthesia and Intensive Care Medicine, Emergency Hospital “Floreasca”, Bucharest, Romania

**Keywords:** Right ventricular function, Mean systemic filling pressure analogue, Portal vein pulsatility index, Systemic congestion, Passive leg raising test, Cardiac surgery

## Abstract

**Background:**

The portal vein pulsatility index (PVPI) reflects systemic congestion and is influenced by both volume status and right ventricular (RV) function. The mean systemic filling pressure analogue (Pmsa), derived from a mathematical model, estimates the interaction between stressed blood volume and systemic vascular compliance, serving as surrogate marker of volume status. This post-hoc analysis of an observational trial investigates the combined role of Pmsa and RV function as determinants of PVPI using echocardiography. Fifty-five mechanically ventilated patients with circulatory failure were included within 6 h of ICU admission following elective open-heart surgery. Fluid-tolerant patients (PVPI < 50%) underwent a passive leg raising (PLR) test; fluid-responsive patients subsequently received 7 mL/kg of Ringer’s lactate. PVPI and Pmsa were measured at five timepoints: baseline (T1), after PLR (T2), upon returning to baseline (T3), after fluid administration (T4), and 20 min post-infusion (T5). RV function parameters, including RV to LV end-diastolic area ratio (RVEDA/LVEDA), tricuspid lateral annular systolic velocity (RV S’), RV fractional area change (RVFAC), pulmonary acceleration time (PAT), and right myocardial performance index (RIMP)—were assessed at T1, T4, and T5. Only fluid-responsive patients were evaluated beyond T3.

**Results:**

At T1, robust multilinear regression including all patients identified RVEDA/LVEDA (β = 10.38; *p* < 0.001), RIMP (β = − 6.54; *p* = 0.002), and RV S’ (β = − 0.60; *p* = 0.002) as significant determinants of squared PVPI. In all patients, repeated measures correlation between Pmsa and PVPI was strong across T1-to-T3 (ρ = 0.785; *p* < 0.001), increasing from a non-significant correlation at T1 (ρ = 0.215; *p* = 0.115). Generalized estimating equations conducted only in fluid-responsive patients across T1, T4, and T5 identified Pmsa (β = 4.19; *p* < 0.001), RV S’ (β = − 5.84; *p* < 0.001), RVEDA/LVEDA (β = 34.85; *p* = 0.018), and RIMP (β = − 35.28; *p* = 0.039) as significant determinants of PVPI.

**Conclusion:**

RV function and Pmsa are key determinants of PVPI. Their combined assessment may support an individualized congestion management by guiding interventions toward volume status, RV function, or both.

*Trial registration* Primary Trial Registration: NCT06440772. Registered 30 May 2024. Retrospectively registered.

**Supplementary Information:**

The online version contains supplementary material available at 10.1186/s13613-025-01498-0.

## Background

Congestion is inconsistently defined, yet central to fluid management [1]. Fluid intolerance, mainly due to tissue congestion but increasingly seen as multifactorial, must be assessed alongside fluid responsiveness, as patients may present with both, complicating clinical decisions [2–5].

Clinically overt tissue congestion, marked by excess interstitial fluid in the lungs or peripheral tissues, can develop even in the absence of hemodynamic congestion, defined by elevated left or right cardiac filling pressures. This dissociation arises because fluid distribution is also governed by factors such as capillary hydraulic conductance and plasma oncotic pressure [6]. Conversely, hemodynamic congestion, though initially subclinical, will inevitably progress to tissue congestion if left unaddressed [7].

Hemodynamic congestion, systemic or central, results from impaired right ventricular (RV)- or left ventricular (LV)-driven blood volume redistribution, further modulated by shifts between stressed and unstressed blood volumes due to dynamic vascular capacitance swings [8–11]. Echocardiography, combined with invasive pressure monitoring, provides a comprehensive, non-invasive method for tracking RV, LV function, and beyond. The mean systemic filling pressure, a surrogate for the balance between stressed blood volume and systemic vascular compliance, can be estimated echocardiographically as the mean systemic filling pressure analogue (Pmsa), using the mathematical modeling technique proposed by Parkin and Leaning [12, 13].

The portal vein pulsatility index (PVPI) is a key marker of systemic hemodynamic congestion. Initially incorporated into the venous excess ultrasound (VExUS) score [14], PVPI has shown potential as an independent predictor, being associated with increased postoperative complications and prolonged life support in high-risk cardiac surgery patients [15]. PVPI is expected to rise either due to absolute volume overload or sympathetically mediated reductions in venous capacitance, both reflected by an increased Pmsa [16–18], or as a result of impaired RV-arterial coupling, whether from reduced RV contractility [19] or elevated RV afterload [20]. However, the extent to which these hemodynamic factors influence PVPI remains unclear, yet understanding this interplay is crucial for its accurate bedside interpretation and implementation.

This post-hoc analysis leverages data from a prospective observational trial that demonstrated post-passive leg raising (PLR) PVPI as a predictor of fluid-induced congestion (PVPI ≥ 50%) in initially fluid-responsive, fluid-tolerant patients, identifying those with reduced RV diastolic reserve [21]. Here, these data are further analyzed to explore the interplay between the hemodynamic determinants of PVPI, including Pmsa and key RV function measures.

## Material and methods

### Study design and ethics

The primary study was a single-center observational investigation conducted between May 2023 and July 2024 at the 1st Department of Cardiovascular Anaesthesia and Intensive Care Medicine, Prof. Dr. C. C. Iliescu Institute for Emergency Cardiovascular Diseases, Bucharest, Romania [21]. The study received approval from the local Institutional Review Board for Biomedical Research (reference number 6066/21 February 2023) and was registered at ClinicalTrials.gov (NCT06440772) on 30 May 2024. This post-hoc analysis follows the STROBE guidelines for observational studies, ensuring transparent and systematic reporting of associations between PVPI, Pmsa, and RV function measures.

### Patient selection

Patients were originally included if they were mechanically ventilated within 6 h of ICU admission following elective open-heart surgery and had acute circulatory failure, defined by clinical signs of hypoperfusion (e.g., mottled skin, oliguria), elevated lactate levels (> 2 mmol/L), reduced central venous oxygen saturation (ScvO₂ < 70%), and an increased central venous-to-arterial carbon dioxide difference (ΔCO₂ > 6 mmHg), with or without hypotension (systolic blood pressure < 90 mmHg or mean arterial pressure (MAP) < 65 mmHg) [21].

All patients were sedated, in sinus rhythm, and mechanically ventilated with central venous and arterial catheters. Ventilator settings included a 5 cmH₂O positive end-expiratory pressure (PEEP), a tidal volume of 6–8 mL/kg ideal body weight, and a fraction of inspired oxygen (FiO₂) adjusted to keep arterial oxygen saturation (SaO₂) at 96–98%, with respiratory rate regulated to maintain arterial carbon dioxide partial pressure (PaCO₂) between 35–40 mmHg [21].

The exclusion criteria were: (1) age under 18 years, (2) conditions interfering with portal vein flow assessment or interpretation (e.g., liver cirrhosis, chronic hepatic disease, suprahepatic/portal vein thrombosis), (3) mechanical circulatory support, (4) cardiac transplant, (5) poor echocardiographic window, and (6) previous amputation [21].

Unlike the original study, which focused on fluid-responsive, fluid-tolerant patients, this post-hoc analysis included all patients for whom data were available, specifically, those who exhibited fluid tolerance at baseline, regardless of fluid responsiveness.

### Echocardiographic data

Transthoracic echocardiography was conducted using a Philips CX50 system with a 2.0–4.0 MHz S4–2 broadband sector array transducer (Koninklijke Philips N.V., Eindhoven, Netherlands) and electrocardiographic gating. A single operator (B.M.) obtained measurements, averaging three consecutive end-expiratory readings, which were later validated by a certified specialist (C.BA.). Sedation was adjusted to a Richmond Agitation-Sedation Scale score of 3 to 4, followed by neuromuscular blockade.

#### Assessment of fluid tolerance and fluid responsiveness

In line with the original study design, all participants were initially screened for fluid tolerance, defined as PVPI < 50%, prior to undergoing further ultrasonographic assessment. Technical details of the measurement process were previously described [21]. Only patients who met the fluid tolerance criterion underwent additional screening for fluid responsiveness, defined as a ≥ 12% increase in left ventricular outflow tract velocity–time integral (LVOT VTI) one minute after passive leg raising (PLR), with values returning to baseline upon resuming the semi-recumbent position.

#### Assessment of cardiac function

Cardiac function data were collected throughout the protocol in accordance with recent recommendations [22]: (1) a measure of LV systolic function, left ventricular ejection fraction (LVEF) assessed via Simpson’s biplane method; (2) a measure of RV enlargement, the ratio of RV to LV end-diastolic areas (RVEDA/LVEDA) assessed via standard apical four chamber view; (3) a tissue Doppler measure of RV longitudinal contraction, tricuspid lateral annular systolic velocity (RV S’); (4) a measure of RV ejection fraction, RV fractional area change (RVFAC) assessed via standard apical four chamber; (5) a measure of pulmonary artery pressure, pulmonary acceleration time (PAT); and (6) a measure of combined systolic and diastolic RV function, right myocardial performance index (RIMP) assessed via tissue Doppler imaging.

#### Assessment of volume state

Pmsa reflects the interaction between stressed blood volume and systemic vascular compliance, serving as a surrogate marker of effective circulating volume [13]. Given the study’s design, echocardiographically-derived Pmsa was a convenient choice, demonstrating strong agreement and correlation with invasive methods, as reported by Yastrebov et al. [12]. The following formula was applied, incorporating MAP, central venous pressure (CVP), and echocardiographically-derived cardiac output (CO) [23]:

$${\text{Pmsa}} = 0.{96}\cdot{\text{CVP}} + 0.0{4}\cdot{\text{MAP}} + {\text{c}}\cdot{\text{CO}},\;{\text{where}}\;{\text{c}} = 0.{96}\cdot0.0{38}\cdot[{94}.{17} + 0.{193}\cdot\left( {{\text{age}}\;{\text{in}}\;{\text{years}}} \right)]/\left\{ {{4}.{5}\cdot0.{99}^{{[\left( {{\text{age}}\;{\text{in}}\;{\text{years}}} \right){-}{15}]}} \cdot0.00{7184}\cdot\left( {{\text{height}}\;{\text{in}}\;{\text{cm}}} \right)^{{0.{725}}} \cdot\left( {{\text{weight}}\;{\text{in}}\;{\text{kg}}} \right)^{{0.{425}}} } \right\}.$$

### Clinical data

In line with the focused scope of this post-hoc analysis, the reported data include demographic information, hemodynamic variables (MAP, CVP, Pmsa), the admission Sequential Organ Failure Assessment (SOFA) score, the preoperative European System for Cardiac Operative Risk Evaluation II (EuroSCORE II), creatinine levels, and perfusion-related variables (arterial lactate, ScvO₂, and ΔCO₂).

### Study protocol

For the purpose of this post-hoc analysis, echocardiographic and clinical parameters necessary for calculating composite measures, such as Pmsa, were analyzed according to the following protocol steps [21]:

- *T1* (Baseline): Baseline measurements included PVPI, LVOT VTI, Pmsa, LVEF, RV and clinical data. Patients who retained fluid tolerance (PVPI < 50%) were further screened for preload reserve (a change in post-PLR LVOT-VTI ≥ 12%).
- *T2* (Post-PLR): One minute after PLR, PVPI, LVOT VTI, and Pmsa were recorded.
- *T3* (Return from PLR): Following the return to the semi-recumbent position, PVPI, LVOT VTI, and Pmsa were recorded after 2 min.
- *T2* and *T3* represented short acquisition windows, during which RV parameters could not be reliably assessed due to time constraints.
- *T4* (Post-Ringer’s Lactate): Among fluid-tolerant patients, those who exhibited preload responsiveness during the T1 to T3 steps received 7 mL/kg of Ringer’s Lactate over 10 min. Two minutes after completing the crystalloid infusion, Pmsa, PVPI, and RV data were recorded.
- *T5* (20 min post-Ringer’s Lactate): Pmsa, PVPI, and RV data were recorded again.

No extra fluids were given, and vasoactive drug regimens were maintained unchanged throughout the T1 to T5 transitions.

### Outcomes

The primary outcome was the relationship between PVPI and its hemodynamic determinants, including Pmsa and RV function echo-derived parameters. Analyses explored these associations at baseline and across multiple time points to assess their stability and predictive value.

### Statistics

Statistical analyses were conducted using NCSS 2024 Statistical Software (NCSS, LLC, Kaysville, Utah, USA), Stata/BE 18.0 (StataCorp, College Station, Texas, USA), and SPSS Statistics for Windows, version 29.0.0.0. (241) (SPSS Inc., Chicago, Ill., USA) as appropriate. The distribution of continuous variables was examined through visual assessment and the Shapiro–Wilk test. Data following a normal distribution are expressed as means with standard deviations (SD), whereas non-normally distributed variables are reported as medians accompanied by interquartile ranges (IQR, 25th–75th percentile). Categorical variables are presented as counts and percentages.

A stepwise approach was applied to identify PVPI determinants. First, a Kruskal–Wallis test assessed differences in Pmsa and RV function parameters across three PVPI strata: Stratum 1 (PVPI ≤ 3.7%, n = 18), Stratum 2 (3.7% < PVPI ≤ 20.0%, n = 20), and Stratum 3 (20.0% < PVPI < 50.0%, n = 17). These strata were generated using NCSS’s tertile-based stratification procedure, which calculated quantile cut-offs from the PVPI distribution at T1. This enabled comparison across increasing levels of systemic congestion while preserving PVPI as a continuous variable. Second, at baseline (T1), a robust multiple linear regression model was used to evaluate the relationship between PVPI, Pmsa, and RV function parameters, incorporating both univariable and multivariable analyses. To reduce the influence of outliers and improve model stability, Tukey’s biweight function (tuning constant 4.685) with 100 iterations was applied. Multicollinearity was checked using variance inflation factors, all of which remained below 3. Third, to explore the dynamic interplay between PVPI and Pmsa over time, a repeated measures correlation using the rmcorr package was conducted across T1 to T3 for all patients, including both fluid-responsive and fluid-unresponsive individuals. Fourth, a generalized estimating equation (GEE) model with exchangeable working correlation matrix was employed to test the longitudinal impact of previously identified predictors on PVPI. This analysis was restricted to fluid-responsive patients with complete data at T1, T4, and T5, since RV function parameters were not recorded beyond T1 and Pmsa beyond T3 for fluid-unresponsive patients. Robust standard errors and Wald Chi-Square statistics were calculated to estimate the strength of each predictor’s contribution.

Sample size calculation was not performed, as this was a post-hoc exploratory analysis with a physiological focus. A *p*-value < 0.05 was considered statistically significant for all tests.

## Results

**Fig. 1:**
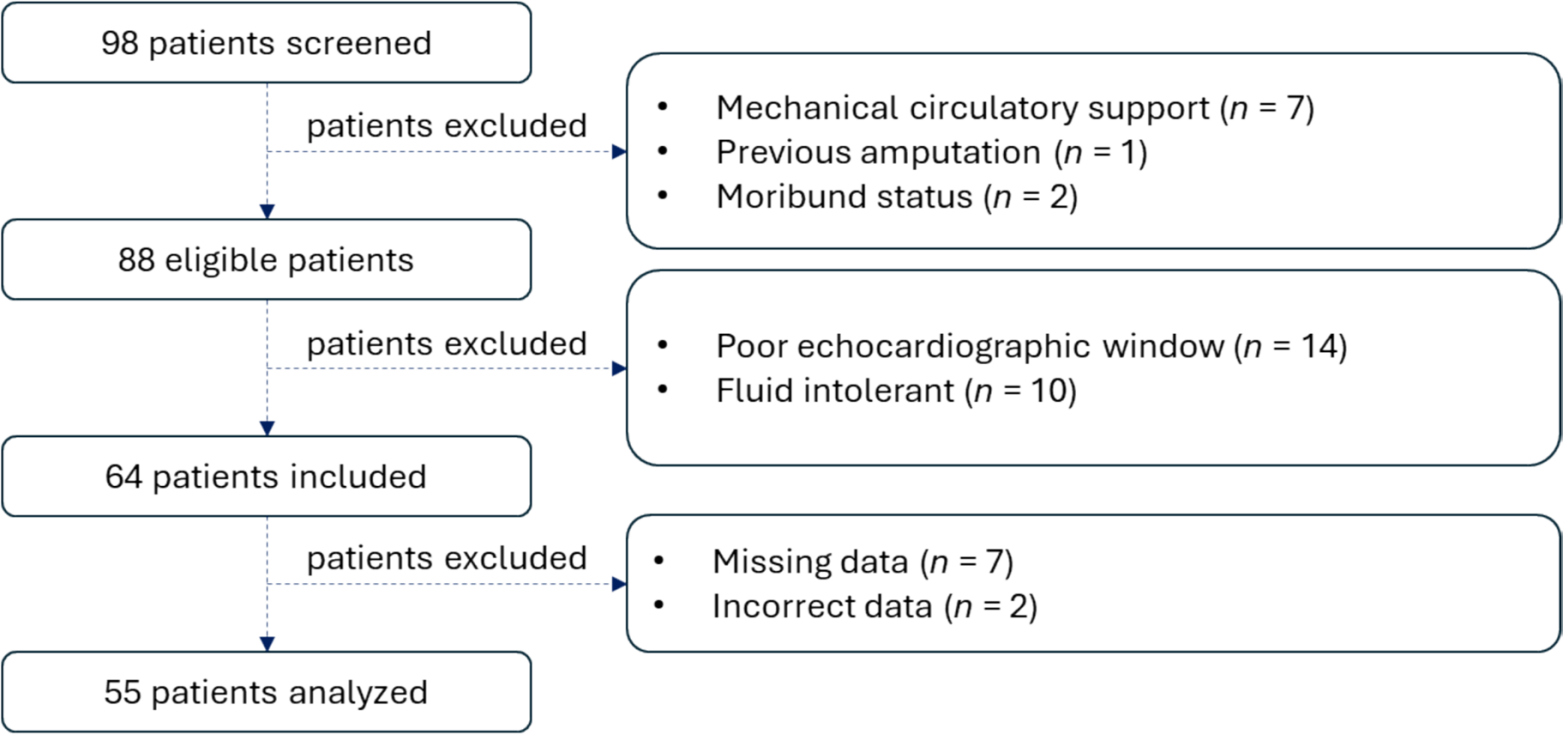
Study Flowchart

Among the 98 patients screened, 64 were included, but 9 were excluded due to missing or incorrect data, resulting in 55 patients for the final analysis (Fig. 1).

Patients had a median age of 63 years, and more than two-thirds were male. The most common interventions were valvular surgery and coronary artery bypass grafting (CABG), with or without concomitant valvular surgery. Two-thirds of all patients were fluid-responsive as defined above and fluid-tolerant at baseline per the inclusion criteria, with a median PVPI of 11% (Table 1).

**Table 1:** Baseline demographic and perioperative data

Parameter	All patients (*n* = 55)
Demographics	
Age, years	63.0 (57.0–68.0)
Gender, male	39 (70.9)
BMI, kg/m^2^	28.6 ± 4.3
Surgery characteristics	
Total duration, min	170.0 (152.0–191.0)
CPB time, min	90.0 (72.0–111.0)
ACC time, min	59.0 (49.0–79.0)
Type of surgery	
Valvular surgery	20 (36.3)
CABG	21 (38.2)
CABG + valvular surgery	4 (7.3)
Wheat/Bentall procedures	6 (10.9)
Miscellaneous	4 (7.3)
Perioperative scores and kidney function	
EuroSCORE II, % mortality	3.4 (2.1–6.7)
SAPS II (6-Hour ICU), % mortality	4.3 (3.1–6.1)
Perfusion variables	
MAP, mmHg	77.1 ± 8.4
CVP, mmHg	7.9 ± 2.6
Pmsa^§^, mmHg	13.9 ± 2.5
Lactate, mmol/L	2.3 (1.8–3.1)
ScVO2, %	69.0 (67.0–74.5)
CI^§^, l/min/m^2^	2.4 (2.0–2.6)
Vasoactive drugs	
Norepinephrine, ng∙kg^−1^∙min^−1^	40.5 (30.0–66.3)
Dobutamine, μg∙kg^−1^∙min^−1^	5.0 (3.0–5.8)
Echocardiographic variables	
LVEF	0.50 (0.45–0.55)
PAT, ms	116.0 (93.0–135.0)
RVFAC	0.31 (0.28–0.38)
RVEDA/LVEDA ratio	0.55 ± 0.13
RV S’, cm/s	9.1 (7.5–10.1)
RIMP	0.42 (0.31–0.49)
PVPI, %	11.0 (2.0–23.0)
Fluid-responsive patients	36 (65.5)

Values are presented as mean ± SD, median (IQR, 25th–75th percentiles) or counts (percentages)

*ACC* aortic cross-clamp, *BMI* body mass index, *CABG* coronary artery bypass graft, *CI* cardiac index, *CPB* cardiopulmonary bypass, *CVP* central venous pressure, *EuroSCORE II* European System for Cardiac Operative Risk Evaluation, *LVEF* left ventricular ejection fraction, *LVEDA* left ventricular end-diastolic area, *MAP* mean arterial pressure, *PAT* pulmonary acceleration time, *Pmsa* mean systemic filling pressure analogue, *PVPI* portal vein pulsatility index, *RIMP* right myocardial performance index, *RV S’* tissue Doppler-derived tricuspid lateral annular systolic velocity, *RVEDA* right ventricular end-diastolic area, *RVFAC* right ventricular fractional area change, *SAPS II* Simplified Acute Physiology Score, *ScVO2* central venous oxygen saturation

^§^These measures are derived using echocardiography

### Assessment of precision

As data were collected by a single operator (B.M.), only intra-observer reproducibility was assessed. Precision was evaluated for PVPI and LVOT VTI. Precision was calculated as 2 × CE, where CE is the coefficient of error defined as CV/√n, with CV being the coefficient of variation (SD/mean of three consecutive measurements) and n = 3 [24]. Median precision was 8.2% (IQR: 7.2–11.8%) for PVPI and 6.3% (IQR: 4.8–7.7%) for LVOT VTI.

### PVPI strata—screening for determinants of PVPI at T1

**Fig. 2:**
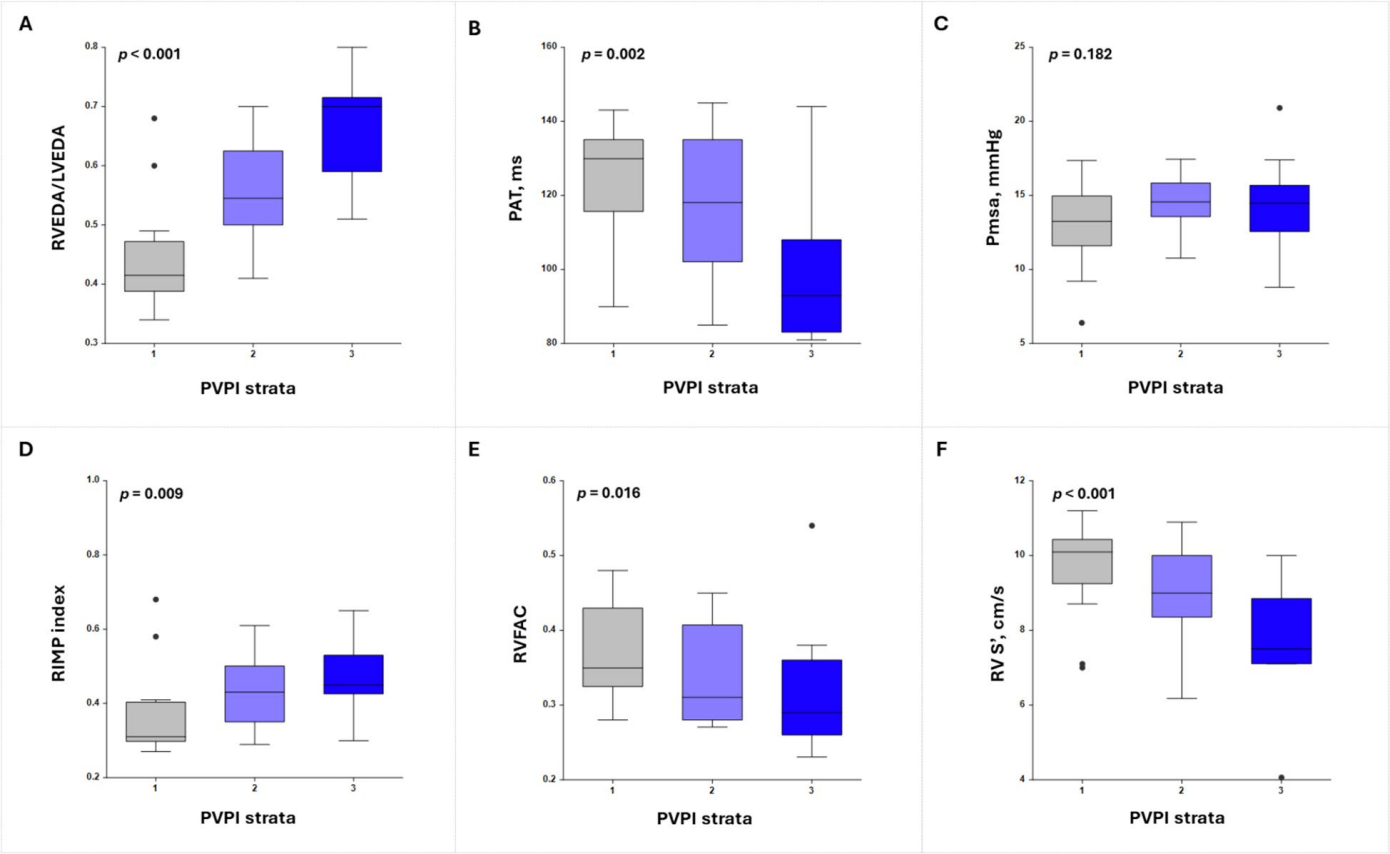
PVPI strata analysis at T1. *PAT* pulmonary acceleration time, *Pmsa* mean systemic filling pressure analogue, *PVPI* portal vein pulsatility index, *RIMP* right myocardial performance index, *RV S’* tissue Doppler-derived tricuspid lateral annular systolic velocity, *RVEDA/LVEDA* right ventricular to left ventricular end-diastolic area ratio, *RVFAC* right ventricular fractional area change, *T1* baseline

All patients were included in this baseline (T1) analysis. A Kruskal–Wallis test was performed to assess variations in Pmsa, RIMP, RVEDA/LVEDA ratio, PAT, RVFAC, and RV S’ across the three PVPI strata. Significant increases were observed in RVEDA/LVEDA (*p* < 0.001) and RIMP (*p* = 0.009), along with significant decreases in PAT (*p* = 0.002), RV S’ (*p* < 0.001), and RVFAC (*p* = 0.016). No significant difference was found for Pmsa across the strata (*p* = 0.182) (Fig. 2). Detailed results, including medians, H-statistics, and *p*-values, are available in the supplementary material (Table S1).

### Regression analysis—integrative assessment of PVPI determinants at T1

All patients were again included in this baseline (T1) analysis. As the Kruskal–Wallis test provides only univariable comparisons, a robust multilinear regression was conducted to assess the independent contribution of each PVPI determinant while adjusting for the others. PVPI was squared to ensure normally distributed residuals. Tests confirmed normality of residuals and absence of multicollinearity, supporting model validity (Tables S2 and S3). Univariable results matched the Kruskal–Wallis findings, showing significance for all factors except Pmsa. In multivariable analysis, only RVEDA/LVEDA, RIMP, and RV S’ remained significant, whereas RVFAC and PAT lost significance. This parsimonious model showed good fit (R^2^ = 0.71, Table 2). Notably, RIMP reversed direction in the multivariable model, becoming inversely associated with PVPI. Although higher RIMP typically reflects worse systolic RV function, this shift suggests that under controlled systolic conditions, RIMP may instead reflect diastolic filling pressures. In contrast, RVEDA/LVEDA and RV S’ maintained the expected associations: larger RV size and reduced longitudinal function were linked to higher PVPI.

**Table 2:** PVPI determinants at T1

Variable	Univariable analysis			Multivariable analysis		
	β	β 95% CI	*p*-value	β	β 95% CI	*p*-value
Pmsa, mmHg	0.20	0.01 to 0.38	0.058	0.13	− 0.01 to 0.26	0.067
PAT, ms	− 0.05	− 0.07 to − 0.03	< 0.001	0.00	− 0.03 to 0.03	0.996
RIMP	5.80	1.99 to 9.61	0.007	− 6.54	− 10.51 to 2.56	0.002
RVEDA/LVEDA	10.89	8.65 to 13.13	< 0.001	10.38	6.14 to 14.61	< 0.001
RVFAC	− 10.49	− 16.85 to − 4.12	0.003	2.31	− 3.47 to 8.10	0.425
RV S’, cm/s	− 0.78	− 1.04 to − 0.52	< 0.001	− 0.60	− 0.97 to 0.22	0.002

Coefficients (β) are presented for each unit increase in squared PVPI

*PAT* pulmonary acceleration time, *Pmsa* mean systemic filling pressure analogue, *PVPI* portal vein pulsatility index, *RIMP* right myocardial performance index, *RV S’* tissue Doppler-derived tricuspid lateral annular systolic velocity, *RVEDA/LVEDA* right ventricular to left ventricular end-diastolic area ratio, *RVFAC* right ventricular fractional area change, *T1* baseline

### Repeated measures correlation—PVPI vs. Pmsa relationship across T1-to-T3

**Fig. 3:**
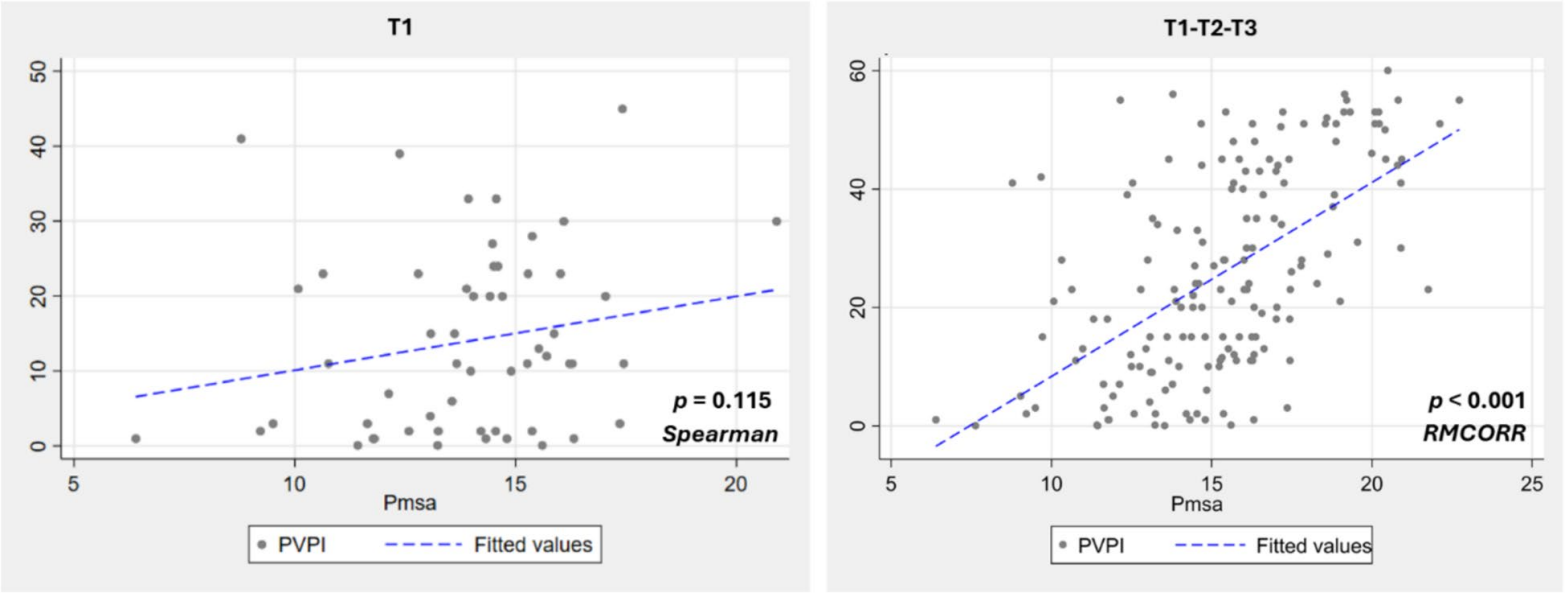
PVPI vs. Pmsa relationship at T1 vs. T1-to-T3. *Pmsa* mean systemic filling pressure analogue, *PVPI* portal vein pulsatility index, *RMCORR* repeated measures correlation, *T1* baseline, *T2* 1 min after passive leg raising, *T3* 2 min after returning to semi-recumbent position

Although Pmsa did not differ significantly across PVPI strata or in the baseline regression analysis, indicating comparable values among patients at T1, we further explored its relationship with PVPI over time. All patients were analysed across T1 (semi-recumbent baseline), T2 (post-PLR), and T3 (return to baseline), a sequence known to alter Pmsa, as demonstrated by Mallat et al. [25] and Guerin et al. [26]. As shown in Table 3 and Fig. 3, the correlation between Pmsa and PVPI strengthened when combining T1–T3, compared to T1 alone. These findings suggest that, while Pmsa is not a baseline determinant of PVPI, it becomes influential under dynamic conditions affecting venous return.

**Table 3:** Dynamic vs. static PVPI–Pmsa relationship

Timepoint	Correlation coefficient (ρ)	ρ 95% CI	*p*-value
T1 (static)	0.215 (Spearman’s ρ)	− 0.056 to 0.460	0.115
T1-to-T3 (dynamic)	0.785 (RMCORR’s ρ)	0.700 to 0.848	< 0.001

*Pmsa* mean systemic filling pressure analogue, *PVPI* portal vein pulsatility index, *RMCORR* repeated measures correlation, *T1* baseline, *T2* 1 min after passive leg raising, *T3* 2 min after returning to semi-recumbent position

### Repeated measures analysis—PVPI determinants across T1, T4 and T5

Repeated measures analysis using a multivariable GEE model was performed at T1, T4, and T5 for all previously identified PVPI determinants (Pmsa, RVEDA/LVEDA, RIMP, and RV S’). The analysis included only fluid-responsive patients, as fluid-unresponsive patients were not assessed beyond T3. Model fit was assessed using the Quasi-likelihood under Independence Model Criterion (QIC = 15,879.462) and Corrected QIC (QICC = 15,872.963), with lower values indicating better fit. As shown in Table 4, all four variables remained significant over time. Pmsa, representing the balance between stressed blood volume and vascular compliance, had the highest Wald Chi-Square value, followed by RV S’, RVEDA/LVEDA, and RIMP—each reflecting RV function. Table S4 presents median and IQR values for all variables across all timepoints and all patients.

**Table 4:** Determinants of PVPI across T1, T4, and T5 in fluid-responsive patients

Variable	β	β 95% CI	Wald Chi-Square	p-value
Pmsa, mmHg	4.19	3.33 to 5.05	91.42	< 0.001
RV S’, cm/s	− 5.84	− 9.03 to − 2.66	12.92	< 0.001
RVEDA/LVEDA	34.85	5.93 to 63.76	5.58	0.018
RIMP	− 35.28	− 68.79 to − 1.76	4.26	0.039

Repeated measures multivariable analysis with coefficients (β) presented for each unit increase in PVPI

*Pmsa* mean systemic filling pressure analogue, *PVPI* portal vein pulsatility index, *RIMP* right myocardial performance index, *RV S’* tissue Doppler-derived tricuspid lateral annular systolic velocity, *RVEDA/LVEDA* right ventricular to left ventricular end-diastolic area ratio, *T1* baseline, *T4* 2 min post-Ringer’s Lactate, *T5* 20 min post-Ringer’s Lactate

## Discussion

This post-hoc analysis supports the physiological concept that systemic congestion, as indicated by PVPI in our study, is primarily determined by two interdependent factors, RV function and intravascular stressed volume (reflected by Pmsa) which interact dynamically over time [8, 10]. Specifically, the RV acts as a *PVPI-setter*, establishing baseline venous congestion, while Pmsa serves as a dynamic *PVPI-modulator*, altering PVPI in response to rapid fluctuations in stressed blood volume. Among the RV parameters analysed, only RV S’, RVEDA/LVEDA, and RIMP were identified as significant determinants. In simpler terms, higher PVPI was associated with reduced longitudinal RV contractility, a more dilated RV, and a prolonged ejection phase relative to the isovolumic phases.

Several considerations need to be addressed in this study. First, the lack of a significant deterministic impact of Pmsa at baseline likely reflects similar baseline Pmsa values across patients, which may result from uniform perioperative fluid management guided by standardized fluid stewardship practices. Additionally, within a certain range of fluid administration, the cardiovascular system exhibits stress relaxation [27], which helps maintain a relatively constant stressed blood volume and, consequently, a stable Pmsa under baseline conditions, outside dynamic perturbations such as postural changes or rapid fluid administration. Second, our analysis confirms that PVPI is influenced by postural changes and fluid loading primarily through shifts in stressed volume, as reflected by Pmsa—an observation supported by previous studies showing that portal flow patterns reliably track fluid balance and responses to decongestive therapies [28–30]. However, systemic congestion, as captured by PVPI, results from a dynamic interplay between blood volume and heart function, which may be further endotyped based on the relative contribution of each factor, an approach associated with distinct clinical outcomes [31]. Third, in a multivariable analysis adjusting for RV function parameters, this study did not identify PAT, a surrogate of RV afterload, as a determinant of PVPI. However, unlike the study by Huette et al. [20], which demonstrated a PVPI increase during an incremental positive end-expiratory pressure trial, our original study was not designed to dynamically manipulate RV afterload and, by extension, RV-arterial coupling. Fourth, this study confirms RV function as a primary determinant of PVPI, consistent with previous findings [15, 32]. Among the RV determinants of PVPI, RIMP exhibited an intriguing and counterintuitive directional change from univariable to multivariable regression analysis. Specifically, while positively associated with PVPI in the univariable model, RIMP became inversely proportional to PVPI in the multivariable model. RIMP is calculated a as the sum of isovolumic relaxation time (IVRT) and isovolumic contraction time (IVCT) divided by ejection time, thereby integrating both systolic and diastolic components. Once systolic function is accounted for in the multivariable model, RIMP primarily reflects only RV diastolic properties. A lower RIMP with increasing PVPI indicates a reduction in its numerator, driven by IVRT shortening under high filling pressures. These elevated pressures contribute to an exhausted splanchnic venous compliance [33], ultimately increasing PVPI. Fifth, our analysis is the first to describe the combined effect of Pmsa and both systolic and diastolic RV function on PVPI. While previous studies reported positive correlations between VExUS and Pmsa [16, 17], our findings underscore the importance of concurrently evaluating RV function. As shown at baseline, similar Pmsa values can result in markedly different PVPI levels depending on RV performance: a well-functioning RV yields lower CVP and PVPI, whereas impaired RV function leads to higher CVP and PVPI [34]. This divergence, recently demonstrated by Ruste et al. [17], is illustrated in Fig. 2 and further supported in the supplementary material (Figure S1 and Table S5), where increasing baseline CVP is observed across the three PVPI strata. Sixth, as a corollary to the fifth consideration, patients with similar PVPI values may require different therapeutic approaches: some may benefit more from decongestive therapy if Pmsa trends high, others from RV optimization when RV dysfunction is evident, and some from a combination of both strategies. In this context, Pmsa serves as a practical and dynamic marker of stressed blood volume [35]. It can be readily calculated at the bedside using the Leaning and Parkin model, as described and referenced in the Methods section, based on three hemodynamic inputs (MAP, CVP, and CO) alongside basic anthropometric variables, using the Excel tool provided in the supplementary material (*S_Pmsa*).

This study has several limitations. First, it is a post-hoc analysis designed to explore a physiological concept, relying on a stepwise evaluation of data due to protocol-defined differences in monitoring of patients caused by the original inclusion criteria. Only fluid-tolerant patients at baseline (PVPI < 50%) were included, and subsequent measurements beyond T3 were restricted to those who were fluid-responsive patients. Also, RV function parameters were measured only at T1, T4, and T5, but not at the dynamic inflection points (T2 and T3). While this limits full dataset uniformity, the physiological insights generated remain relevant and plausibly extendable to patients with PVPI ≥ 50% and fluid-unresponsive profiles. Second, although this is a physiological study involving numerous echocardiographic measurements, intra-observer reproducibility was confirmed only for the primary measurements reflecting congestion (PVPI) and flow (LVOT VTI). Nevertheless, all measurements were performed by a single experienced operator, eliminating inter-observer variability and supporting measurement consistency. Third, the post-hoc design inherently carries the risk of bias, although the prospective nature of the original trial and the pre-specified physiological framework may mitigate some of these concerns. Finally, this was a single-center pilot study with a small sample size, which may affect the generalizability and statistical power of some findings.

## Conclusion

This study identifies RV function and Pmsa as independent determinants of PVPI. Their combined assessment may assist clinicians in determining the optimal strategy to mitigate congestion, whether by targeting volume status, RV performance, or both.

## Supplementary Information

Supplementary Material 1.

Supplementary Material 2.

## Data Availability

The datasets used and/or analyzed during the current study are available from the corresponding author upon reasonable request.

## Abbreviations

ACC: Aortic cross-clamp
BMI: Body mass index
CABG: Coronary artery bypass graft
CI: Cardiac index
95% CI: 95% Confidence interval
CPB: Cardiopulmonary bypass
CVP: Central venous pressure
Eh: Global heart efficiency
EuroSCORE II: European System for Cardiac Operative Risk Evaluation II
GEE: Generalized estimating equation
IVCT: Isovolumic contraction time
IVRT: Isovolumic relaxation time
LVEF: Left ventricular ejection fraction
LVEDA: Left ventricular end-diastolic area
LVOT-VTI: Left ventricular outflow tract velocity time integral
MAP: Mean arterial pressure
PAT: Pulmonary acceleration time
Pmsa: Mean systemic filling pressure analogue
PVPI: Portal vein pulsatility index
ρ (RHO): Correlation coefficient
RIMP: Right myocardial performance index
RMCORR: Repeated measure correlation
RVEDA: Right ventricular end-diastolic area
RVFAC: Right ventricular fractional area change
RV S’: Tissue Doppler-derived tricuspid lateral annular systolic velocity
SAPS II: Simplified Acute Physiology Score II
ScVO2: Central venous oxygen saturation
T1: Baseline
T2: 1 Minute after passive leg raising
T3: 2 Minutes after returning to the semi-recumbent position
T4: 2 Minutes post-Ringer’s Lactate
T5: 20 Minutes post-Ringer’s Lactate
