# Supplementary material for: Decoding portal vein pulsatility: hemodynamic determinants in a post-hoc analysis of a prospective observational trial
Source: Ann Intensive Care. 2025 Jun 14;15:81. doi: 10.1186/s13613-025-01498-0 (PMC12167214; doi:10.1186/s13613-025-01498-0)
Supplement: Supplementary file 1 — Supplementary Material 1. [file 13613_2025_1498_MOESM1_ESM.docx]

**Supplementary Material**

**Tables S**

**Table S1.** PVPI strata analysis with the Kruskal-Wallis test – T1

| **Variable** | **Stratum 1**  **PVPI ≤ 3.7%** | **Stratum 2**  **3.7% < PVPI ≤ 20.0%** | **Stratum 3**  **20% < PVPI < 50.0%** | **H-Value** | ***p*-Value** |
| --- | --- | --- | --- | --- | --- |
| RVEDA/LVEDA | 0.42 (0.39 – 0.47) | 0.55 (0.50 – 0.63) | 0.70 (0.59 – 0.72) | 29.804 | < 0.001 |
| PAT, ms | 130.0 (115.8 -135.0) | 118.0 (102.0 – 135.0) | 93.0 (83.0 – 108.0) | 12.942 | 0.002 |
| Pmsa, mmHg | 13.2 (11.6 – 14.9) | 14.6 (13.6 – 15.8) | 14.5 (12.6 – 15.7) | 3.408 | 0.182 |
| RIMP | 0.31 (0.30 – 0.40) | 0.43 (0.35 – 0.50) | 0.45 (0.43 – 0.53) | 9.441 | 0.009 |
| RVFAC | 0.35 (0.33 – 0.43) | 0.31(0.28 – 0.41) | 0.29 (0.26 – 0.36) | 8.333 | 0.016 |
| RV S’, cm/s | 10.1 (9.3 – 10.4) | 9 (8.4 – 10.0) | 7.5 (7.1 – 8.9) | 16.118 | < 0.001 |

Values are presented as median (IQR, 25^th^ -75^th^ percentiles)*.* *Abbreviations:* PAT, pulmonary acceleration time; Pmsa, mean systemic filling pressure analogue; PVPI, portal vein pulsatility index; RIMP, right myocardial performance index; RV S’, tissue Doppler-derived tricuspid lateral annular systolic velocity; RVEDA/LVEDA, right ventricular to left ventricular end-diastolic area ratio; RVFAC, right ventricular fractional area change; T1, baseline.

**Table S2.** Multicollinearity report for robust multilinear regression analysis – T1

| **Variable** | **VIF** | **R^2^ vs Others** | **Tolerance** |
| --- | --- | --- | --- |
| RVEDA/LVEDA | 2.74 | 0.64 | 0.36 |
| PAT, ms | 2.72 | 0.63 | 0.37 |
| Pmsa, mmHg | 1.16 | 0.14 | 0.86 |
| RIMP | 2.38 | 0.58 | 0.42 |
| RVFAC | 1.60 | 0.37 | 0.63 |
| RV S’, cm/s | 2.65 | 0.62 | 0.38 |

All variance inflation factors (VIFs) were below 3 and tolerances above 0.3, indicating no concerns regarding multicollinearity. *Abbreviations:* PAT, pulmonary acceleration time; Pmsa, mean systemic filling pressure analogue; PVPI, portal vein pulsatility index; R², coefficient of determination from regressing one predictor on all others; RIMP, right myocardial performance index; RV S’, tissue Doppler-derived tricuspid lateral annular systolic velocity; RVEDA/LVEDA, right ventricular to left ventricular end-diastolic area ratio; RVFAC, right ventricular fractional area change.

**Table S3.** Residual normality tests

| **Test Name** | **H0: Residuals Normally Distributed** | | |
| --- | --- | --- | --- |
|  | Test Statistic Value | *p*-Value | Reject H0 at α = 0.2? |
| Shapiro-Wilk | 0.99 | 0.915 | No |
| Anderson-Darling | 0.15 | 0.965 | No |
| D'Agostino Skewness | 0.89 | 0.378 | No |
| D'Agostino Kurtosis | -0.34 | 0.732 | No |
| D'Agostino Omnibus | 0.90 | 0.640 | No |

**Table S4.** Variables and analyses across timepoints

| **Analysis** | **All Patients (fluid-responsive and fluid-unresponsive)**  **(n = 55)** | | | **Fluid-responsive Patients**  **(n = 36)** | |
| --- | --- | --- | --- | --- | --- |
|  | **T1** | **T2** | **T3** | **T4** | ***T5*** |
| Kruskal-Wallis | Yes | No | No | No | No |
| Robust regression | Yes | No | No | No | No |
| RMCORR | Yes | Yes | Yes | No | No |
| GEE regression | Yes | No | No | Yes | Yes |
| **Variables included in the analysis** | | | | | |
| RVEDA/LVEDA | 0.55 (0.43 – 0.67) | - | - | 0.60 (0.50 – 0.71) | 0.53 (0.45 – 0.69) |
| PAT, ms | 116.0 (93.0 – 135.0) | - | - | 109.5 (90.0 – 128.0) | 118.0 (96.5 – 131.8) |
| RIMP | 0.42 (0.31 -0.49) | - | - | 0.46 (0.35 – 0.53) | 0.40 (0.30 – 0.49) |
| RVFAC | 0.31 (0.28 – 0.38) | - | - | 0.30 (0.27 – 0.35) | 0.34 (0.29 – 0.38) |
| RV S’, cm/s | 9.1 (7.5 – 10.1) | - | - | 8.8 (7.4 – 9.7) | 9.0 (7.9 – 10.1) |
| Pmsa, mmHg | 14.3 (12.6 – 15.5) | 17.8 (15.3 – 20.0) | 15.6 (13.3 – 16.4) | 16.0 (14.5 -18.6) | 14.5 (13.0 – 15.9) |
| PVPI, % | 11.0 (2.0 -23.0) | 41.0 (24.0 – 52.0) | 28.0 (15.0 – 43.0) | 47.5 (21.8 – 56.0) | 28.5 (13.5 – 41.8) |

Values are presented as median (IQR, 25^th^ -75^th^ percentiles)*. Abbreviations:* GEE, generalized estimating equation; PAT, pulmonary acceleration time; Pmsa, mean systemic filling pressure analogue; PVPI, portal vein pulsatility index; RIMP, right myocardial performance index; RMCORR, repeated measures correlation; RV S’, tissue Doppler-derived tricuspid lateral annular systolic velocity; RVEDA/LVEDA, right ventricular to left ventricular end-diastolic area ratio; RVFAC, right ventricular fractional area change; T1, baseline; T2, 1 minute after passive leg raising; T3, 2 minutes after returning to semi-recumbent position; T4, 2 minutes post-Ringer’s Lactate; T5, 20 minutes post-Ringer’s Lactate.

**Table S5.** Extended PVPI strata analysis with Kruskal-Wallis test – T1

| **Variable** | **Stratum 1**  **PVPI ≤ 3.7%** | **Stratum 2**  **3.7% < PVPI ≤ 20.0%** | **Stratum 3**  **20% < PVPI < 50.0%** | **H-Value** | ***p*-Value** |
| --- | --- | --- | --- | --- | --- |
| CVP, mmHg | 6.5 (5.0 – 9.0) | 8.0 (8.0 – 9.8) | 9.0 (6.5 – 10.0) | 6.516 | 0.038 |

Values are presented as median (IQR, 25^th^ -75^th^ percentiles)*. Abbreviations:* CVP, central venous pressure; Pmsa, mean systemic filling pressure analogue; PVPI, portal vein pulsatility index; T1, baseline.

**Figures S**

**
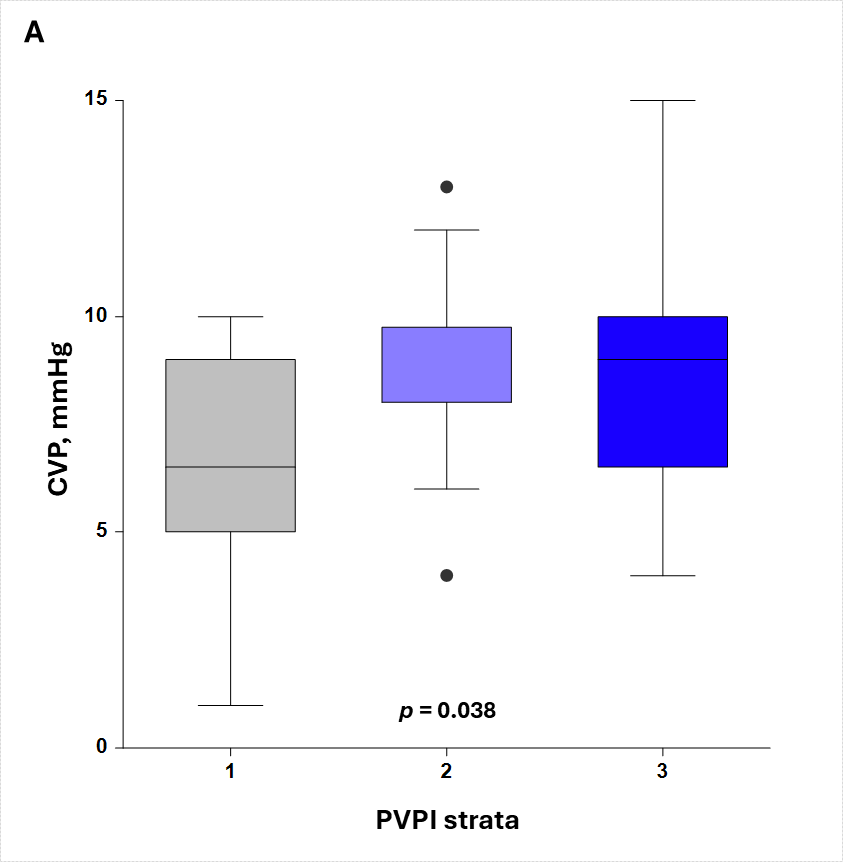
**

**Figure 1S.** Extended PVPI strata analysis at T1. *Abbreviations:* CVP, central venous pressure; PVPI, portal vein pulsatility index; T1, baseline.
